# Recording the tactile P300 with the cEEGrid for potential use in a brain-computer interface

**DOI:** 10.3389/fnhum.2024.1371631

**Published:** 2024-06-12

**Authors:** M. Eidel, M. Pfeiffer, P. Ziebell, A. Kübler

**Affiliations:** Institute of Psychology, University of Würzburg, Würzburg, Germany

**Keywords:** brain-computer interface (BCI), P300-event-related potential, tactile P300, tactually evoked potentials, somatosensory sensitivity

## Abstract

Brain-computer interfaces (BCIs) are scientifically well established, but they rarely arrive in the daily lives of potential end-users. This could be in part because electroencephalography (EEG), a prevalent method to acquire brain activity for BCI operation, is considered too impractical to be applied in daily life of end-users with physical impairment as an assistive device. Hence, miniaturized EEG systems such as the cEEGrid have been developed. While they promise to be a step toward bridging the gap between BCI development, lab demonstrations, and home use, they still require further validation. Encouragingly, the cEEGrid has already demonstrated its ability to record visually and auditorily evoked event-related potentials (ERP), which are important as input signal for many BCIs. With this study, we aimed at evaluating the cEEGrid in the context of a BCI based on tactually evoked ERPs. To compare the cEEGrid with a conventional scalp EEG, we recorded brain activity with both systems simultaneously. Forty healthy participants were recruited to perform a P300 oddball task based on vibrotactile stimulation at four different positions. This tactile paradigm has been shown to be feasible for BCI repeatedly but has never been tested with the cEEGrid. We found distinct P300 deflections in the cEEGrid data, particularly at vertical bipolar channels. With an average of 63%, the cEEGrid classification accuracy was significantly above the chance level (25%) but significantly lower than the 81% reached with the EEG cap. Likewise, the P300 amplitude was significantly lower (cEEGrid R2–R7: 1.87 μV, Cap Cz: 3.53 μV). These results indicate that a tactile BCI using the cEEGrid could potentially be operated, albeit with lower efficiency. Additionally, participants’ somatosensory sensitivity was assessed, but no correlation to the accuracy of either EEG system was shown. Our research contributes to the growing amount of literature comparing the cEEGrid to conventional EEG systems and provides first evidence that the tactile P300 can be recorded behind the ear. A BCI based on a thus simplified EEG system might be more readily accepted by potential end-users, provided the accuracy can be substantially increased, e.g., by training and improved classification.

## 1 Introduction

Electroencephalography (EEG) is a versatile and accessible tool to record brain activity non-invasively from the scalp and is therefore widely used in psychology, neuroscience and medicine. One field of application particularly relevant for the present study are EEG based brain-computer interfaces (BCIs), a technology that allows users to interact with the environment without requiring muscular control.

In a BCI, brain activity is acquired with EEG or similar technology and translated into commands by specialized machine learning algorithms (for a review, see Lotte et al., 2018). This allows access to a variety of functions, often for communication and device control (Vaughan, 2003; Kübler, 2019; Saha et al., 2021). Many BCI paradigms rely on event-related potentials (ERPs) such as the P300 as input signal to determine the user’s intent (Polich and Margala, 1997; McFarland, 2020). P300 BCIs are based on the oddball-paradigm (Donchin et al., 1978), in which the user has to concentrate on rare and randomly occurring “target” stimuli while ignoring frequent “non-target” stimuli. Target stimulus rarity and unexpectedness have long been considered important factors to elicit large P300 amplitudes in the oddball paradigm, though this notion has recently been challenged (Verleger and Śmigasiewicz, 2016; Wascher et al., 2020). Generally, P300 amplitude and location may vary between individuals, which might be due to numerous factors, for example the subject’s age (van Dinteren et al., 2018).

The P300 is typically measured at electrode positions Fz, Cz and Pz. It appears in the EEG as a positive deflection about 300 ms after the target stimulus (Polich, 2007). Thus, it can be used to automatically detect which stimulus was attended by the user, which is the basis of many BCI approaches.

Due to their independence from voluntary motor control, BCIs have the potential to be a valuable assistive tool to restore or enhance functions even for patients with severe motor paralysis (Kaufmann et al., 2013; McCane et al., 2014; Botrel et al., 2017; Eidel et al., 2021). Potential BCI end-users, thus, include people diagnosed with the *locked-in syndrome* (LIS), a state of near-complete paralysis, which can manifest in patients with *amyotrophic lateral sclerosis*, stroke, or other conditions (Bauer et al., 1979; Wolpaw et al., 2002; Smith and Delargy, 2005).

Although there is a considerable need for innovative devices to assist patients with severe or even total motor paralysis in their daily lives, prospective end-users rarely get the chance to establish BCIs in their own home (with a few notable exceptions; for an overview see Sellers et al., 2010; Kübler, 2019; Allison et al,. 2020). Thus, a notable translational gap remains, despite the fact that BCIs have long been proven feasible under laboratory conditions (Kübler, 2013; Kübler et al., 2015; Nijboer, 2015). The user-centered approach to BCI design has been adapted to evaluate BCI operation in a multimodal way (Zickler et al., 2011). It includes measures of efficiency (accuracy) and effectiveness (speed in relation to accuracy) of a BCI, but also the individual user satisfaction (Choi et al., 2017; Han et al., 2022). Low user satisfaction has been suggested as a major contributor to the translational gap, as BCI users often report that they were not satisfied with certain practical aspects of the EEG hardware. Frequent remarks include that setting up the EEG was too difficult for caregivers, the EEG cap cumbersome with too many cables, and that the EEG gel in the hair was unpleasant (Kübler et al., 2014; Lynn et al., 2016; Eidel and Kübler, 2020). On top of that, the EEG cap is often considered as too visually prominent and aesthetically unappealing (Zickler et al., 2011; Hoon Lee et al., 2014).

**Figure 1:**
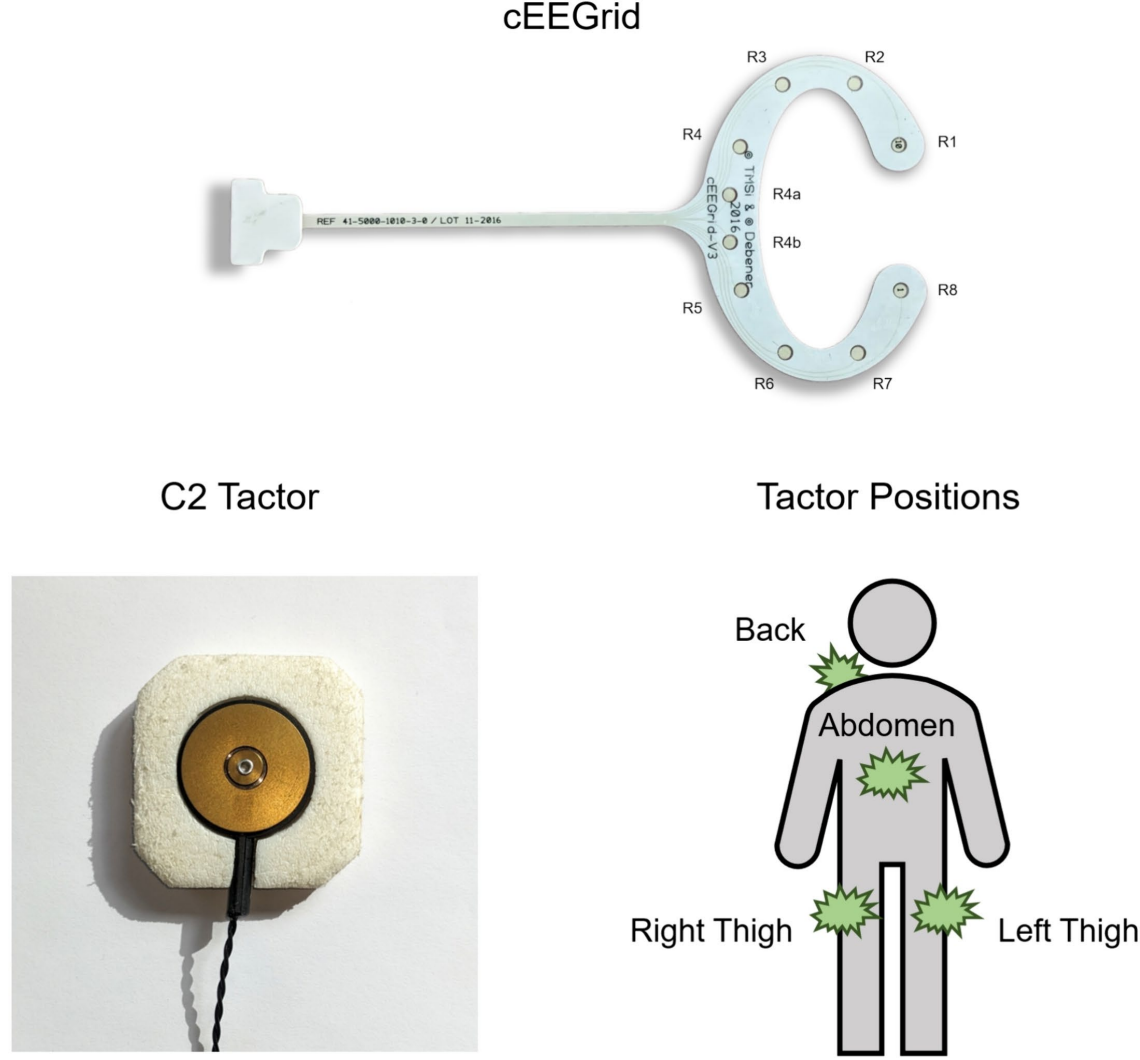
Hardware setup. On the right ear cEEGrid (version V3), channels R4a and R4b serve as ground and reference, respectively. Left ear channels are named analogously (L1, L2 etc.). Also shown is a single C2 tactor (Engineering Acoustic Inc., Casselberry, United States) and the four tactor positions. To facilitate stimulus perception, two devices were attached per position (activated synchronously).

Notably, there are efforts to alleviate some of these issues, for example by developing alternative and more compact EEG systems. An extensive study recently confirmed that the auditory N100, MMN, P300 and N400 could be recorded reliably with electrodes placed around the ears (Meiser and Bleichner, 2022), despite some expected signal loss as compared to standard scalp-EEG positions. In fact, specialized ear-EEG systems already exist: Examples include tiny EEG sensors that are placed into the *concha* and outer ear canal (Looney et al., 2011; Kidmose et al., 2013) as well as devices that are positioned around or behind the ear (Do Valle et al., 2014), particularly the *cEEGrid* (Debener et al., 2015). The cEEGrid is a semi-disposable, flex-printed sensor array of ten Ag/AgCl electrodes per ear (Figure 1 shows the channel configuration for the right ear). With its c-shape, it is simply attached around the ear with double-sided adhesive tape. Only a small amount of gel is added onto the electrode surfaces to ensure low impedances. This gel is protected from drying out by the tape seal around it. Indeed, test subjects wore cEEGrids for several hours of normal activity outside of the lab with no substantial decline in recording quality (Debener et al., 2015). A cEEGrid system is flexible and can be connected wirelessly, allowing for high mobility while being relatively unobtrusive and discreet.

Multiple studies have already described how ERPs, for instance the N100, P100 and P300, can be recorded and reliably classified using the cEEGrid – sometimes with accuracies statistically on par with those from an EEG cap (Debener et al., 2015; Bleichner et al., 2016; Denk et al., 2018). Notably, it was found that classification of several ERPs worked best at bipolar channels, in particular from electrode pairs which have a large inter-electrode distance and are arranged toward the origin of the signal of interest (Bleichner et al., 2016). These studies used either visual or auditory stimulation to elicit the P300. To our knowledge, a tactile P300 has never been recorded with the cEEGrid.

Thus, with the present study, we aimed at providing a first demonstration of a cEEGrid in combination with our tactile BCI system (Kaufmann et al., 2014), which was originally developed for potential wheelchair control by paralyzed users. Tactile and other non-visual P300 paradigms (e.g., auditory BCIs) have already been shown to be feasible with conventional scalp EEG recording (Furdea et al., 2009; Brouwer and van Erp, 2010; Schreuder et al., 2011; Hill and Schölkopf, 2012; Riccio et al., 2012; Kaufmann et al., 2014; Simon et al., 2015; Baykara et al., 2016; Halder et al., 2016). Importantly, tactile and auditory BCIs could pose a valuable alternative for end-users with impairment of eyesight or gaze control, who may not be able to operate visual paradigms efficiently (Kübler and Birbaumer, 2008; Brunner et al., 2010). Moreover, several studies confirmed the tactile BCI modality was feasible for severely impaired, potential end-users with LIS (Severens et al., 2014), in one example even during a long-term study at the patient’s own home (Eidel et al., 2021). Although the performances of tactile P300 BCIs are usually lower than those of visual paradigms (see Eidel and Kübler, 2020 for an overview), we have repeatedly found that training led to significant performance increases among healthy participants (Herweg et al., 2016; Eidel and Kübler, 2020; Ziebell et al., 2020). Latest evidence further suggested that somatosensory sensitivity correlated with accuracy and with performance increases across multiple sessions (Eidel and Kübler, 2022).

To summarize, many crucial preconditions for successful use of a BCI as an assistive tool for patients have been met, but the translational gap remains. It appears that the frequent requests (e.g., Zickler et al., 2011) for a simpler, less cumbersome and less visible alternative to the EEG cap might be fulfilled with the cEEGrid, such that BCIs may be more readily implemented and accepted by potential end-users.

With this study, we aimed to explore whether the cEEGrid constitutes a potential option for the home use of a tactile BCI. First, however, we need to further validate the cEEGrid as a device for potential tactile P300 BCI control. Thus, we focused on assessing its feasibility in an application focused, tactile BCI scenario built around established BCI software, and compared it directly to an established cap EEG.

This approach was guided by several hypotheses: Based on numerous observations from other modalities, we expected that the tactile P300 can be recorded with the cEEGrid and that ERP amplitudes correlate between cEEGrid and scalp EEG (H1).

Secondly, we hypothesized that cEEGrid classification accuracy in a typical BCI setting would be significantly above chance level (H2a) or even above the 70% criterion for useful BCI control (Kübler et al., 2001) (H2b). We further expected that classification accuracy would not be significantly lower as compared to an EEG cap (H3) (Bleichner et al., 2016).

Additionally, we sought to confirm previous observations of a correlation between somatosensory sensitivity and classification accuracy (H4).

## 2 Methods

### 2.1 Participants

Forty healthy participants (P) were invited and completed one BCI session. However, two had to be excluded from analysis due to bad recording quality at key cEEGrid positions. Another participant was excluded because he reported that he did not fully concentrate on the tactile stimuli.

The final sample, thus, comprised *n* = 37 participants (27 female, 10 male). The age of the participants ranged between 21 and 59 years (*M* = 24.6, SD = 6.2). From three of the participants (P13, P16, and P32), a single run each was excluded from cEEGrid analysis due to technical issues during the recording. All participants except P1 had no prior experience with a tactile BCI. Participants gave written informed consent to the procedure and received either a monetary compensation of € 10/h or course credits. The study was approved by the ethical review board of the Institute of Psychology at the University of Würzburg, Germany (GZEK 2013-11) and conducted in accordance with the ethical guidelines of the Declaration of Helsinki.

### 2.2 General session procedure

Participants were seated in front of a monitor and set up with tactile stimulation devices. Firstly, they performed a brief somatosensory intensity discrimination task. After EEG preparation, participants were given an abbreviated demonstration run of the paradigm. This was done to give participants an opportunity to get used to the unfamiliar tactile stimulation and confirm that they understood the procedure, so that they could fully concentrate on the task during the first actual run.

Participants then performed six tactile P300 runs with short breaks in between. During a run, all tactor positions were activated multiple times in a random sequence. Participants were instructed to concentrate only on the current target position indicated on the monitor (e.g., by silently counting the target vibrations) and to ignore all other stimuli. They were advised to avoid unnecessary blinking and other muscular activity. During a run, the target position (either front, back, left or right) was pseudo-randomly switched after ten vibrations of each tactor. Each body position was the target twice per run, without immediate repetitions of the same position. Participants received no online feedback of results. After six runs, this process resulted in a total of 480 target and 1,440 non-target trials.

### 2.3 Stimulation

Vibrotactile stimulation was applied with tactor devices (C2 tactors; Engineering Acoustic Inc., Casselberry, United States) which translated the stimulation provided by BCI2000 into short vibrations of 220 ms (vibration frequency: 250 Hz, inter-stimulus interval: 400 ms). Tactors were positioned at right and left thighs, abdomen and upper back of the participants, as in previous studies based on this paradigm (e.g., Kaufmann et al., 2014; Eidel and Kübler, 2020), since it was assumed that this setup facilitated stimulus discrimination due to the relatively large distances between these body positions (Brouwer and van Erp, 2010). Figure 1 shows a single C2 tactor and an overview of all tactor positions on the body. To minimize possible confounding effects, the tactors were adjusted until participants reported no perceivable intensity differences between the four tactor positions.

### 2.4 Intensity discrimination

To test whether the participants’ individual tactile sensitivity had a predictive function on BCI performance, we implemented a simple intensity discrimination task at the beginning of the session, as described in Eidel and Kübler (2022) (similar approaches were used in Nagarajan et al., 1998; Mahns et al., 2006).

Briefly, participants received an array of various two-stimulus trials and had to report whether they perceived an intensity difference between the two stimuli. A first reference stimulus was set to 100% intensity, whereas the second could be decreased in 5% steps. Stimuli pairs were separated by a pause of 500 ms. Pre-recorded tactor noises were presented over speakers, such that the participants could not hear the actual sounds from the tactor device.

The intensity of a trial was set pseudo-randomly. Typically, very similar or extremely different second stimuli were reliably reported as such. In between, however, there were intensity ranges in participants’ responses were inconsistent after several repetitions of the same trial. Those intensities were tested more frequently. Hence, several responses across all stimulus intensities were processed to determine a threshold value at which a participant could not tell whether the second stimulus intensity was equal or lower as compared to the reference.

**Figure 2:**
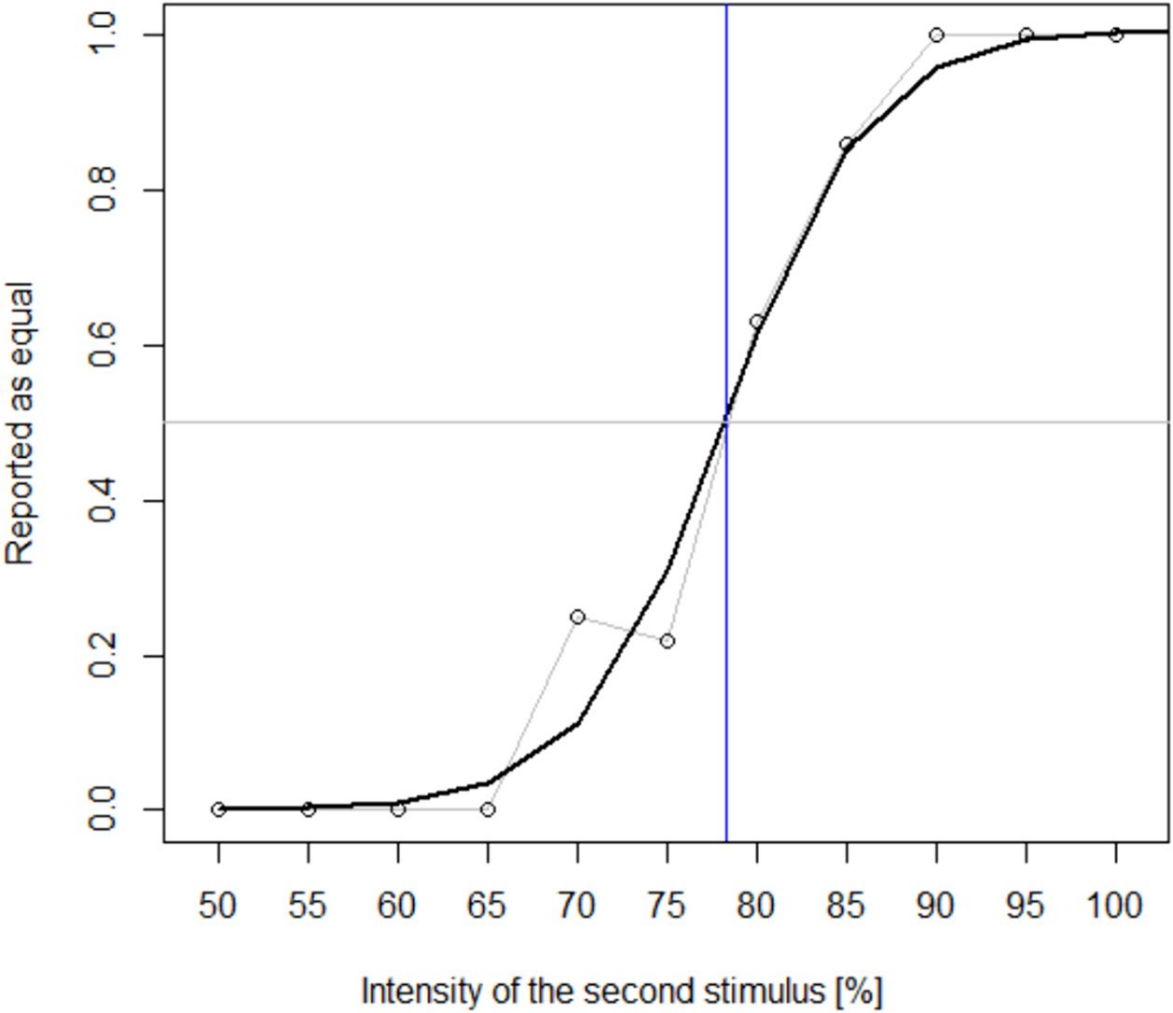
Example of a response curve from the tactile discrimination task (data from P3). A sigmoidal fit was used to estimate the point at which the second stimulus was reported as equal in 50% of trials. The respective stimulus intensity (here at 78%) was extracted as the participant’s threshold for analysis.

To calculate a sensitivity metric, each participant’s response ratio for each stimulus intensity was projected onto a scale from 0 (always reported as unequal) to 1 (always reported as equal). A sigmoidal function was automatically fit to these data (see Figure 2), and the intensity value at the response ratio of 0.5 was extracted.

The sensitivity threshold was thus defined as the intensity value (of the second stimulus) that was perceived as equal in 50% of trials. The thresholds were subjected to a Pearson correlation test with the participants’ offline accuracies.

### 2.5 EEG recording and preprocessing

Scalp EEG was recorded from twelve passive Ag/AgCl electrodes at positions Fz, FC1, FC2, C3, Cz, C4, P3, Pz, P4, O1, Oz, and O2, with ground and reference electrodes placed at the left and right earlobes. For the cEEGrid system, the area around both ears was cleaned thoroughly with alcohol swabs and abrasive gel. A small drop of electrolyte gel was added onto the electrode surfaces before both cEEGrids were attached. The cEEGrids are semi-disposable. If one was being reused, double sided stickers were placed around the electrodes for better adhesion. Both cEEGrids were connected to an amplifier with an adapter cable.

Impedances at the scalp electrodes were kept below 5 kΩ and below 30 kΩ at the cEEGrid (Debener et al., 2015). If cEEGrid impedances were too high, a syringe with a blunt metal tip was used to add more gel without removing the cEEGrid if possible (as a result, 32 participants had values below 15 kΩ at all positions).

The signal from both EEG systems was digitized with two separate BrainAmp amplifiers (Brain Products, Gilching, Germany) and received by the BrainVision Recorder software. These data were relayed to BCI2000 (Schalk et al., 2004) via the remote data access protocol and stored using a resolution of 250 Hz and a 48–52 Hz notch filter. BCI2000 handled the paradigm control (i.e., randomization and presentation of stimuli) and the recording of EEG data and markers. Since BCI2000 has no native support for tactor control, a Python 2.7 UDP script was used to address the tactor’s API (provided by the manufacturer).

No online classification was performed, as two different EEG systems were involved in the recording. Providing feedback from a combination of EEG data, but also from just one of the EEG systems might have confounded our observations. Instead, we focused on offline analysis for both systems separately, without extensive pre-processing of the data, as an indicator of potential online performance.

For analysis of the ERP physiology, EEG data were bandpass filtered (0.1 to 30 Hz). For the following analysis, vertical bipolar channels were calculated for the cEEGrid. Bipolar combinations of ear channels have been analyzed thoroughly (Debener et al., 2015; Bleichner et al., 2016; Meiser and Bleichner, 2022), and certain combinations are known to approximate positions on the scalp (da Silva Souto et al., 2021).

Data from both EEG systems were then split into epochs of 800 ms post-stimulus, plus an additional 100 ms pre-stimulus period for baseline correction. Epochs were rejected as artefacts if they contained any value above or below 75 μV. Valid epochs were averaged separately into target and non-target groups for visual analysis and extraction of physiological features. All offline processing was performed with MATLAB^©^ (R2015b).

### 2.6 EEG analysis

Both EEG systems were evaluated and compared based on two metrics: Target classification accuracy (offline), and ERP amplitude (visual and quantitative analysis).

For quantitative analysis of the ERP, the *mean target amplitudes,* calculated from a specific time window, were extracted from each participant’s target epoch average. For the scalp EEG, analysis was focused on a time window of 350–650 ms (post-stimulus) at positions Fz, Cz and Pz.

ERP analysis for the cEEGrid was focused on the vertical bipolar channels R1–R8, R2–R7, R3–R6, R4–R5 and their left counterparts (Bleichner et al., 2016), which are known to extrapolate toward the classical central scalp positions (highest P300 amplitudes in our data were recorded at Cz) (da Silva Souto et al., 2021).

Initial analysis revealed that the ERPs at positions behind the ear had a slightly higher latency. The time window for amplitude extraction was thus shifted to 400–700 ms post-stimulus.

Offline accuracy was calculated for the two EEG systems separately with a leave-one-out cross-validation approach to prevent overfitting. Classification with either EEG system was based on all available electrodes (cap: 12, cEEGrid: 18) plus the eight bipolar channels in case of the cEEGrid. It was performed with the stepwise linear discriminant analysis classifier included in BCI2000.

Accuracies from the two EEG systems were subjected to a two-tailed paired *t*-test. To detect differences in the EEG physiology, a repeated measures ANOVA of the mean amplitudes was calculated on the EEG channels of interest. If the assumption of sphericity was violated, Greenhouse–Geisser adjusted results are reported. Finally, we ran a Pearson correlation test between the two channels with the highest ERP mean amplitude of either EEG system (Pacharra et al., 2017).

## 3 Results

### 3.1 ERP comparison

**Figure 3:**
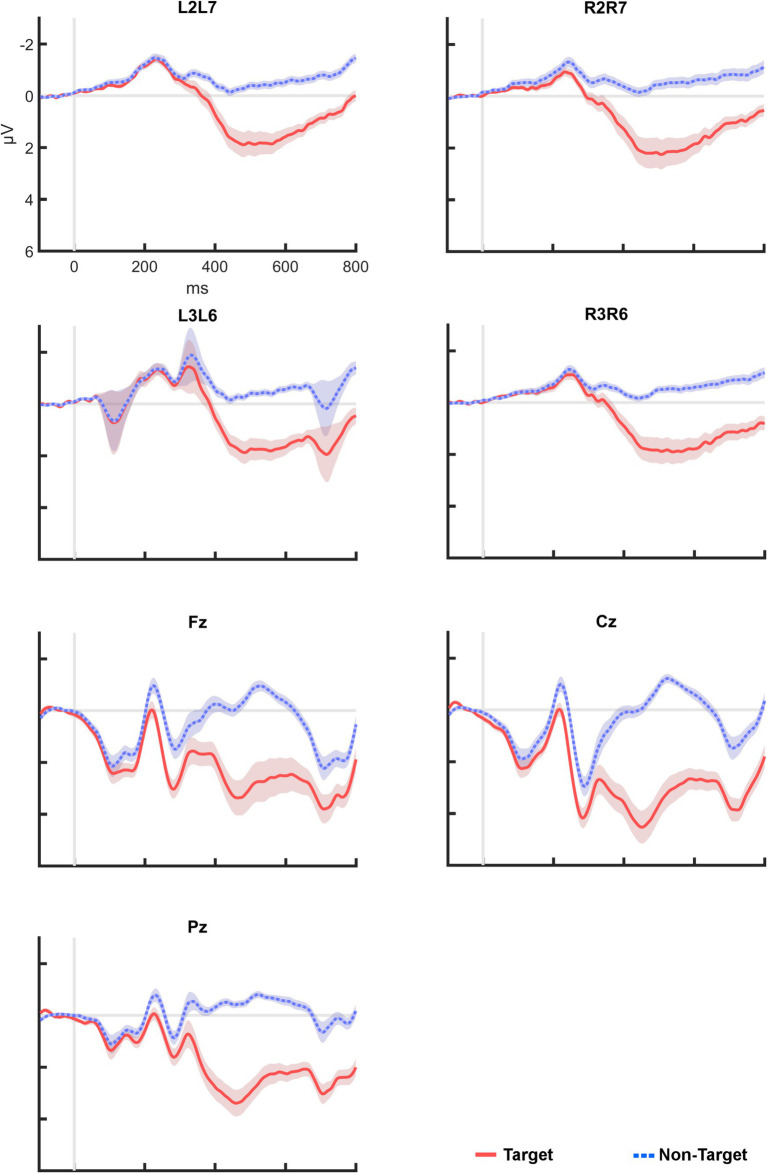
Post-stimulus epochs averaged across all participants. All plots are displayed with the same scale. Shaded areas indicate standard error between participants. ERPs on the bipolar cEEGrid channels were clearly visible, but smaller than at Fz, Cz, and Pz.

Cap EEG data from P15 was excluded from analysis due to an abundance of electromagnetic stimulation artefacts originating from the tactors. Initial assessment indicated that for the cEEGrid, the recorded ERP was indeed highest at the vertical bipolar channels, in particular at those with the highest inter-electrode distance. Thus, the following analysis focused on L2–L7, L3–L6, R2–R7, and R3–R6. Figure 3 shows the grand averages of post-stimulus epochs from these channels in comparison to Fz, Cz and Pz. Visual analysis revealed a distinctive positive deflection in the P300 range. In the cEEGrid, the ERP peak appeared slightly later (*M* = 501 ms post-stimulus) than at the central midline positions (*M* = 463 ms).

Table 1 shows the mean amplitudes extracted from the channels of interest. Mean amplitudes recorded from the EEG cap reached up to *M* = 3.53 μV (SD = 2.92) at Cz, and from the cEEGrid up to 1.87 μV (SD = 2.72) at R2–R7. Mean amplitudes at the cEEGrid channels were generally lower than at the central midline electrodes.

**Table 1:** Mean amplitudes obtained from the two EEG systems.

System	Position	Mean amplitude [μV] (SD)
EEG Cap	Fz	2.70 (3.61)
	**Cz**	**3.53** (2.92)
	Pz	2.68 (2.36)
cEEGrid	**R2–R7**	**1.87** (2.72)
	R3–R6	1.69 (2.49)
	L2–L7	1.58 (2.11)
	L3–L6	1.63 (2.06)

Bold values indicate the highest value per electrode system.

A repeated measures ANOVA revealed a significant effect of electrode position on ERP amplitude (*F*_(2.96, 103.6)_ = 13.20, *p* < 0.001, *η_p_^2^* = 0.274). Bonferroni-corrected post-hoc comparisons indicated a significant difference between Cz and all four bipolar channels of the cEEGrid. For both Fz and Pz, we found a significant amplitude difference to L2–L7, L3–L6, and R3–R6. Amplitudes at R2–R7, the cEEGrid channel with the highest mean values, were not significantly different to Fz and Pz (*p* > 0.05).

Comparison of channels within the same electrode system (e.g., Fz vs. Cz vs. Pz), revealed no significant difference between scalp electrodes or any combination of the bipolar cEEGrid positions (*p* > 0.05).

**Figure 4:**
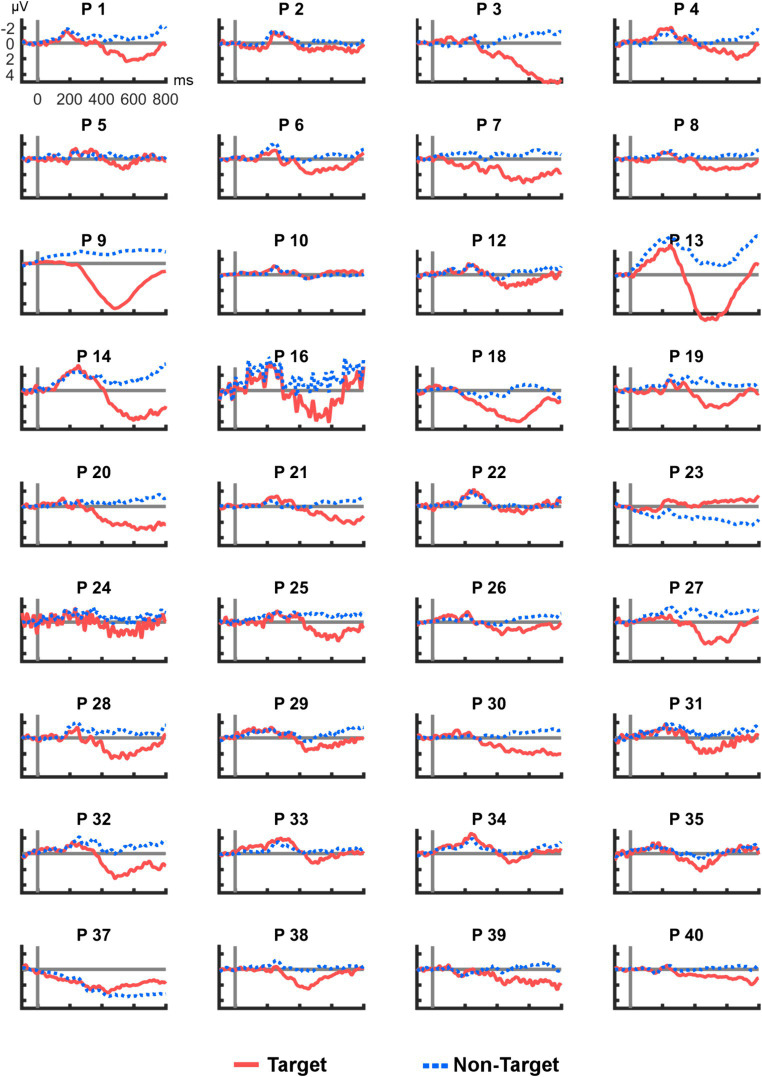
Average post-stimulus epochs from R2–R7 for each participant. ERP polarity was inversed for P23 and P37.

**Figure 5:**
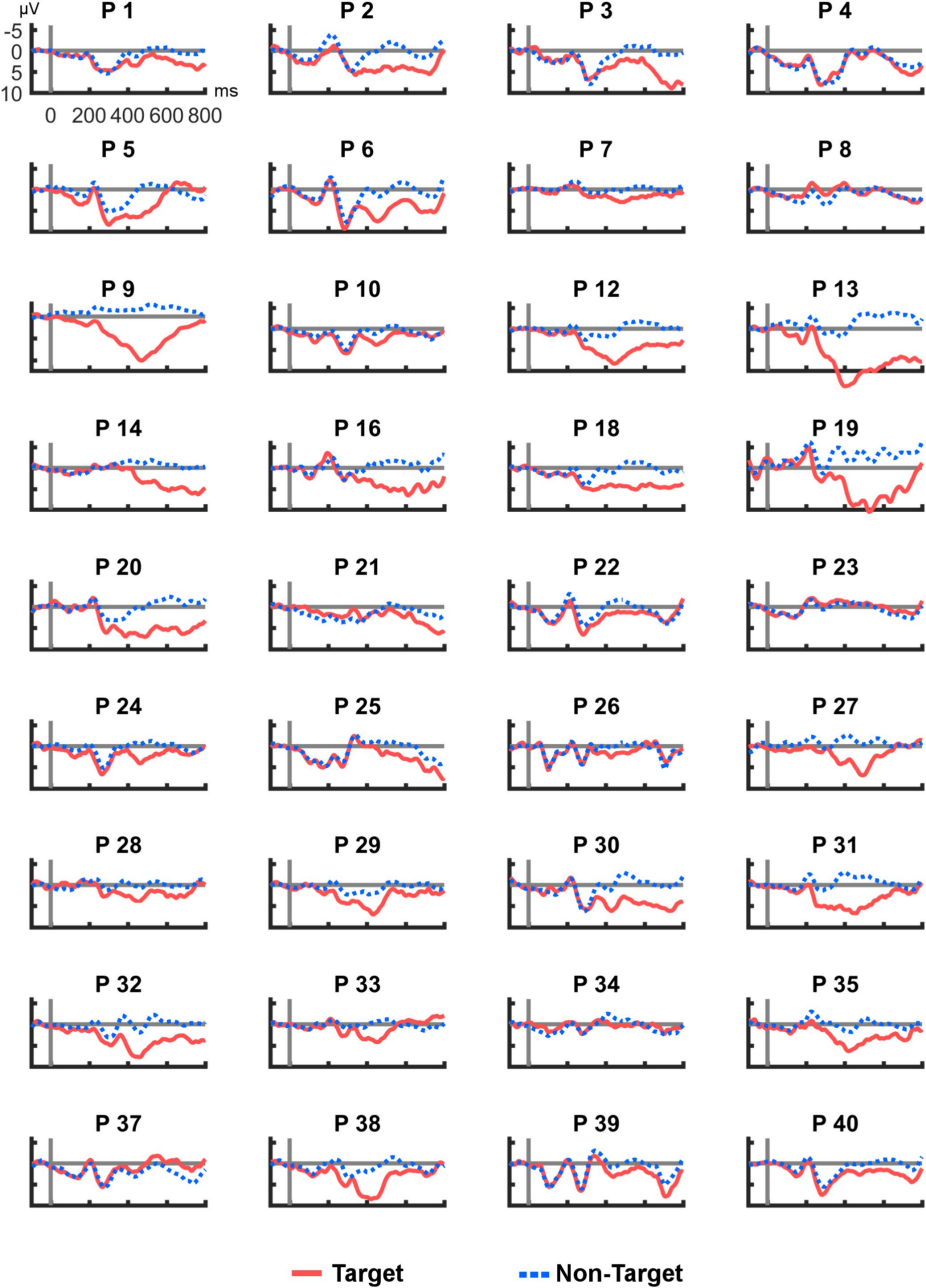
Average post-stimulus epochs for each participant at Cz. As in the cEEGrid data, ERP polarity of P37 was inversed.

Figure 4 shows all ERP averages from channel R2–R7 per participant. This visualization reveals high heterogeneity in the individual responses to the tactile stimuli: Some participants appeared to have no clear deflection in the P300 range (e.g., P10), others showed a particularly high peak (e.g., P9). In two cases (P23 and P37), a deflection with a negative polarity was found. For comparison, Figure 5 provides the same overview for position Cz, where similar observations were made (note that different scaling was used in Figures 3–5 for better visibility). Finally, Pearson correlation between the mean amplitudes of Cz and R2–R7 was significant (*r_p_* = 0.753, *p* < 0.001).

### 3.2 Target classification

The cross-validated offline accuracies were very heterogeneous. Individual averages ranged from 6 to 98% for the cEEGrid and from 42 to 100% for the scalp EEG (see Table 2 for an overview). Thus, all scalp EEG accuracies were above the threshold of approximately 37% (Billinger et al., 2013; Combrisson and Jerbi, 2015) to be considered to be significantly (α = 0.05) above the chance level of 25%. This threshold was also reached with the cEEGrid system for all but three participants (P10, P34, and P35). The usability criterion of at least 70% (Kübler et al., 2001) was met by 27 participants with the scalp EEG and 14 participants with the cEEGrid.

**Table 2:** Overview of offline accuracies (% correct responses) and sensitivity thresholds (percent intensity) per participant.

Participant	EEG Cap [%]	cEEGrid [%]	Sensitivity [%]
1	77	83	83.4
2	92	75	82.6
3	91	79	78.2
4	83	65*	69.6
5	96	90	71.8
6	83	65*	76.2
7	73	50*	65.0
8	65*	48*	73.5
9	100	98	76.2
10	77	29^+^	77.3
12	100	69*	92.0
13	96	85	59.3
14	65*	65*	73.9
15	–	44*	92.5
16	67*	67*	77.7
18	92	52*	56.8
19	100	90	85.5
20	79	73	–
21	56*	44*	79.3
22	96	40*	78.4
23	50*	46*	75.3
24	92	85	65.8
25	42*	46*	–
26	71	42*	80.9
27	79	85	88.8
28	79	67*	74.5
29	94	56*	65.2
30	90	69*	76.8
31	100	96	89.4
32	95	75	76.9
33	96	56*	74.8
34	50*	6^+^	74.8
35	75	23^+^	61.3
37	54*	38*	73.9
38	96	71	69.6
39	69*	56*	73.3
40	94	81	58.4
M	81 (SD = 17)	63* (SD = 21)	74.6 (SD = 9.0)

Scalp EEG data from P15 was contaminated by several artefacts and thus not analyzed. ^+^Not significantly above chance level; ^*^ below usability criterion (Kübler et al., 2001).

With a mean of 63% (SD = 21), cEEGrid accuracies were significantly lower than accuracies calculated from the scalp electrodes (*M* = 81%, SD = 17) (paired *t*-test, *p* < 0.001). Nonetheless, Pearson correlation between accuracies of both EEG systems was high and significant (*r_p_* = 0.647, *p* < 0.001).

### 3.3 Intensity discrimination

Somatosensory sensitivity thresholds were extracted based on the data from the intensity discrimination task. Table 2 provides an overview of the determined sensitivity thresholds per participant. As in our last study (Eidel and Kübler, 2022), some participants quickly revealed a distinct threshold, whereas the responses of other participants were less consistent, necessitating more trials (on average, 37 ± 8.7 trials were applied). The equal/unequal responses from P20 were still too inconsistent to calculate a threshold, despite performing 53 trials, and were excluded from the analysis. P25 was excluded since no sounds could be played to mask the tactor buzzing due to technical problems with the speakers.

On average, the sensitivity threshold was determined at a second-stimulus intensity of *M* = 74.6% (at this intensity, participants correctly detected the difference of the stimuli in 50% of trials). Guided by hypothesis H4, we calculated a Pearson correlation between the discrimination thresholds and offline accuracies obtained from both the scalp EEG and the cEEGrid. However, no significant correlation could be revealed in either case (both *p* > 0.1). We ran an exploratory analysis for potential correlations of the discrimination thresholds with the mean ERP amplitudes from both systems but found no significant effects.

## 4 Discussion

### 4.1 ERP elicitation (H1)

Analysis of the EEG physiology from scalp positions verified that a tactile P300 was successfully elicited with this paradigm. In the time window of interest, average amplitudes were highest at Cz (*M* = 3.53 μV), slightly exceeding the respective values from our previous studies (2.73 μV, Eidel and Kübler, 2020; 2.95 μV, Eidel and Kübler, 2022).

There was a notable inter-subject variability of P300 amplitudes, which might be due to a variety of factors, including motivation (Kleih et al., 2010) and age (van Dinteren et al., 2018), as well as somatosensory sensitivity and target discrimination difficulty (Comerchero and Polich, 1999; Eidel and Kübler, 2022).

As expected, the highest amplitudes in the cEEGrid system were found at the vertical bipolar channels (up to 1.87 μV at R2–R7) (Bleichner et al., 2016; da Silva Souto et al., 2021). At these channels we found a smaller deflection (as compared to the scalp), which we interpreted as the P300. The significant and relatively high correlation (*r_p_* = 0.753) of mean amplitudes between scalp EEG and cEEGrid supported this interpretation.

These two observations – reduced cEEGrid amplitudes, but a notable correlation to scalp EEG data – are well in line with previous ERP studies with the cEEGrid (e.g., Debener et al., 2015; Pacharra et al., 2017). Smaller ERP amplitudes at the cEEGrid were often observed in the literature, possibly due to the distance from the areas of their physiological origin, the small distance between the bipolar electrodes, or because the bipolar combination does not perfectly match the 10–20 position of interest (Bleichner et al., 2016; Garrett et al., 2019). Moreover, the signal-to-noise ratio (SNR) of the P300 and other ERPs were generally lower at the cEEGrid in comparison to cap measurements (Pacharra et al., 2017). A decrease at of the cEEGrid SNR was also reported for subcortical auditory potentials (Garrett et al., 2019). However, some signal loss is to be expected when recording certain ERPs at ear positions, even with conventional electrodes (Meiser and Bleichner, 2022). Overall, though the method of extrapolation using the bipolar combinations of ear channels has been shown to be useful, it appears that it cannot always compensate for the distance to the signal source.

The P300 at the cEEGrid might also have been affected by factors specific to the tactile paradigm. For instance, while the visual P300 amplitude is often largest over parietal regions (Ravden and Polich, 1999; Kaufmann et al., 2011), in the tactile modality highest amplitudes are often measured at central or even frontal electrode positions (Thurlings et al., 2012; van der Waal et al., 2012; Kaufmann et al., 2014; Severens et al., 2014; Herweg et al., 2016; Eidel and Kübler, 2020). These positions might be less accessible for the cEEGrid, which could further explain the reduced amplitudes behind the ear.

Overall, we found strong evidence to support hypothesis H1 and, thus, provide first evidence that the tactually evoked P300 can be recorded by the cEEGrid.

### 4.2 BCI performance (H2–H3)

Offline accuracies were calculated to provide an indicator of potential online performance of the cEEGrid in comparison to the conventional scalp EEG recording.

The average cEEGrid accuracy of 63% was significantly above the chance level, which confirmed our prediction H2a (while also lending further support for H1, predicting P300 elicitation). Unfortunately, the usability criterion of 70% was not reached on average, such that H2b was not confirmed on the group level. Still, 39% of participants met or exceeded this threshold and could potentially operate a tactile BCI with the cEEGrid.

Comparing both systems, we found that accuracies at the cEEGrid were significantly lower, mirroring our observations from the EEG physiology. Hypothesis H3 must, thus, be rejected. This was in contrast to other studies which reported no significant accuracy differences between cEEGrid and scalp EEG (e.g., Bleichner et al., 2016). The distance of the cEEGrid from relevant generators of the tactually evoked P300 may account for this difference in accuracy.

In any case, the high correlation between the two systems suggests that both captured the same physiological process (Bleichner et al., 2016).

A closer look at the performance calculated from scalp positions revealed that accuracies (*M* = 81%) were well above both chance level and the 70%-criterion. This value was very close to the online performance of 79% (session one) reported in our previous study which used the same paradigm (Eidel and Kübler, 2022). This also indicates successful replication, which is an important side effect in light of the replication crisis (Shrout and Rodgers, 2018).

Interestingly, in the study by Bleichner et al. (2016), above-chance classification results were achieved by 85% (scalp EEG) and 80% (cEEGrid) of participants. With the present paradigm, although cEEGrid classification accuracy was significantly lower as compared to the scalp EEG, the above-chance rate was achieved by 92% of participants (100% for scalp EEG data). Although it is hard to compare these different paradigms, the relatively high above-chance rate seems encouraging, as it shows that a meaningful information transfer may be possible for most users.

Accuracy achieved with the tactile P300 BCI has been shown to improve with training (Eidel and Kübler, 2022). We hypothesize that such improvement would also occur when using the cEEGrid, though this must be tested in further studies with healthy and physically impaired participants alike. Further improvement might also be possible by utilizing more advanced classification algorithms (Lotte et al., 2018). Future studies should identify which algorithms perform best with the specific design of the cEEGrid.

### 4.3 Somatosensory sensitivity (H4)

The average discrimination threshold was determined at 74.6%, which was almost identical to the value from our last study (74.9% in session one, Eidel and Kübler, 2022). In contrast to the results of the prior study, however, we found no significant correlation between the somatosensory sensitivities and (offline) accuracies. Hypothesis H4 could, thus, not be supported.

The role of somatosensory sensitivity therefore remains inconclusive, since the previously observed correlation with accuracy could not be reproduced. Still, it may play an important role for training effects of the tactile paradigm (as shown in Eidel and Kübler, 2022), but this could not be analyzed in the single-session design of the present study and awaits further investigation.

## 5 Significance and conclusion

Our study adds to the growing list of literature that compares ear-centered EEG with more conventional EEG setups (e.g., Mikkelsen et al., 2015; Bleichner et al., 2016; Pacharra et al., 2017; Garrett et al., 2019). The main goal of this study was to describe the cEEGrid system’s capability to record the P300 when elicited with an existing tactile BCI paradigm. Hence, the study was designed for best comparability of the EEG systems in a potential online BCI scenario, but at this stage, not for speed or optimization of classification algorithms.

Overall, we found clear evidence that the P300 can be measured with the cEEGrid and that the obtained epochs could be feasibly classified with a widely used BCI machine learning algorithm. However, both ERP amplitudes and classification accuracies were significantly lower as compared to the simultaneously recorded scalp EEG. Since multiple studies demonstrated that the accuracy of the tactile paradigm can be significantly improved across several sessions, even a brief training program might alleviate this performance issue. If higher performances can be achieved, the small and flexible cEEGrid system would constitute a promising tool to increase home application among patients with severest motor impairment up to the locked-in or complete locked-in syndrome, who might accept it more readily than a conventional cap EEG system.

## Data availability statement

The raw data supporting the conclusions of this article will be made available by the authors, without undue reservation.

## Ethics statement

The study involving humans were approved by the Ethical Review Board of the Institute of Psychology at the University of Würzburg, Germany. The studies were conducted in accordance with the local legislation and institutional requirements. The participants provided their written informed consent to participate in this study. Written informed consent was obtained from the individual(s) for the publication of any potentially identifiable images or data included in this article.

## Author contributions

ME: Conceptualization, Formal analysis, Investigation, Methodology, Software, Writing – original draft, Writing – review & editing. MP: Conceptualization, Formal analysis, Investigation, Writing – review & editing. PZ: Conceptualization, Writing – review & editing. AK: Funding acquisition, Project administration, Writing – review & editing, Supervision.
